# Visualization of mandibular movement relative to the maxilla during mastication in mice: integration of kinematic analysis and reconstruction of a three-dimensional model of the maxillofacial structure

**DOI:** 10.1186/s12903-021-01879-4

**Published:** 2021-10-14

**Authors:** Go Yasuda, Emi Moriuchi, Ryo Hamanaka, Ayumi Fujishita, Tomoko Yoshimi, Kana Yamamoto, Kaori Hayashida, Yoshiyuki Koga, Noriaki Yoshida

**Affiliations:** 1 Department of Orthodontics and Dentofacial Orthopedics, Graduate School of Biomedical Sciences, Nagasaki University, 1-7-1 Sakamoto, Nagasaki, 852-8588 Japan; 2 Department of Orthodontics, Nagasaki University Hospital, 1-7-1 Sakamoto, Nagasaki, 852-8588 Japan

**Keywords:** Motion capture, Jaw movements, Mastication, Mouse, Rigid transformation, EMG

## Abstract

**Background:**

Mastication is one of the most fundamental functions for the conservation of human life. To clarify the pathogenetic mechanism of various oral dysfunctions, the demand for devices for evaluating stomatognathic function has been increasing. The aim of the present study was to develop a system to reconstruct and visualize 3-dimensional (3D) mandibular movements relative to the maxilla, including dynamic transition of occlusal contacts between the upper and lower dentitions during mastication in mice.

**Methods:**

First, mandibular movements with six degrees of freedom were measured using a motion capture system comprising two high-speed cameras and four reflective markers. Second, 3D models of maxillofacial structure were reconstructed from micro-computed tomography images. Movement trajectories of anatomical landmark points on the mandible were then reproduced by integrating the kinematic data of mandibular movements with the anatomical data of maxillofacial structures. Lastly, 3D surface images of the upper dentition with the surrounding maxillofacial structures were transferred to each of the motion capture images to reproduce mandibular movements relative to the maxilla. We also performed electromyography (EMG) of masticatory muscles associated with mandibular movements.

**Results:**

The developed system could reproduce the 3D movement trajectories of arbitrary points on the mandible, such as incisor, molars and condylar points with high accuracy and could visualize dynamic transitions of occlusal contacts between upper and lower teeth associated with mandibular movements.

**Conclusions:**

The proposed system has potential to elucidate the mechanisms underlying motor coordination of masticatory muscles and to clarify their roles during mastication by taking advantage of the capability to record EMG data synchronously with mandibular movements. Such insights will enhance our understanding of the pathogenesis and diagnosis of oral motor disorders by allowing comparisons between normal mice and genetically modified mice with oral behavioral dysfunctions.

## Background

Mastication is one of the most fundamental functions for the conservation of human life. Nevertheless, the pathogenesis of various oral dysfunctions has not been adequately clarified due to the complex mechanisms involved in mastication and jaw muscle coordination. Oral motor behaviors have previously been investigated using animal models such as rabbits [1], hamsters [2], guinea pigs [3], cats [4, 5], rats [6, 7], and mice [8–12] to elucidate the central and peripheral mechanisms controlling masticatory function.

Recent developments in molecular biological techniques have allowed the creation of issue-specific knockout mouse [13–17] and several mutant mice, e.g., osteopetrotic (op/op) mice [18, 19] and serotonin receptor-deficient mice [20]. Serotonin is a monoaminergic neurotransmitter that is broadly distributed throughout the brain, and is suggested to have something to do with oral dyskinesias [21], similar to Parkinson’s disease [22, 23], Huntington’s disease [24–26] and hemiballism [27, 28] that often result from dysfunction of the basal ganglia. Demand for mice to use as animal models of oral dyskinesia is likely to increase.

Comparisons between normal mice and genetically modified mice with behavioral dysfunctions in the maxillofacial region can help elucidate the etiologies of motor disorders in the craniomandibular system.

Given this background, our previous study described the development of an optoelectronic device for measuring mandibular movements with six degrees of freedom, allowing analysis of 3-dimensional (3D) movements of arbitrary points on the mandible in mice [12]. That system could visualize movement trajectories of the mandible, but not its motions relative to the maxilla, such as dynamic occlusal contacts between the upper and lower dentitions during mastication.

The purpose of the present study was to develop a system to measure mandibular movements with six degrees of freedom and to visualize dynamic occlusal relationships between the upper and lower dentitions as well as to analyze 3D movement trajectories of arbitrary points on the mandible by integrating kinematic data obtained from a motion capture system with geometric data of the maxillofacial structure created from micro-computed tomography (micro-CT) images during mastication in mice. In addition, the proposed device was equipped with an electromyography (EMG) system to record activities of masticatory muscles synchronously with mandibular movements.

## Methods

The system consists of five steps: (1) measurement of mandibular movements, (2) recording of masticatory muscle activities, (3) reconstruction of the 3D anatomical model, (4) reproduction of mandibular movements, (5) reproduction of mandibular movements relative to the upper dentition and visualization of dynamic occlusion. Steps (1), (3) and (4) have been tested and described in detail in a separate article [12]. The experimental protocol of this study was reviewed and approved beforehand by the Animal Welfare Committee of Nagasaki University based on the Animal Care Standards of this institution (approval no. 1812181496-2). This study conformed the ARRIVE guidelines.

**Fig. 1:**
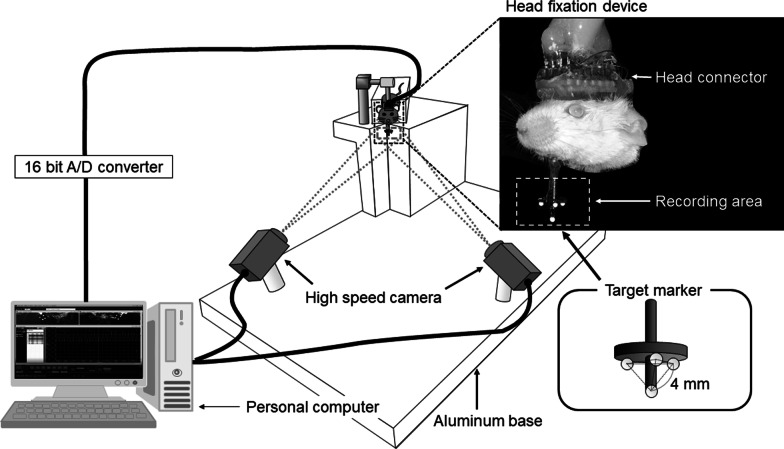
Schematic illustration of the optoelectronic jaw-tracking system. Two high-speed cameras are positioned orthogonally toward the target markers. Four markers are attached to the mandible of a mouse whose head movements have been restrained using the head fixation device

Figure 1 shows a schematic representation of the system for measuring mandibular movements with six degrees of freedom using a motion capture system (DIPP-Motion V3D; DITECT). Four markers were attached to the mandible of a subject mouse (Jcl:ICR mouse; Clea, Tokyo, Japan), and two high-speed cameras (HAS220; DITECT, Tokyo, Japan) captured the movements of each marker at a frame rate of 200 frames/s.

**Fig. 2:**
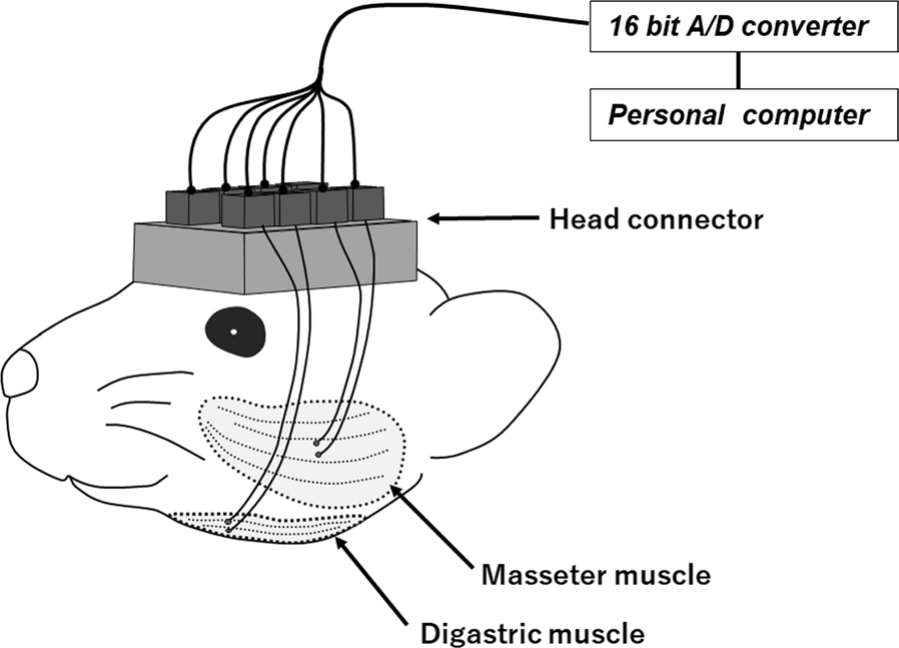
Schematic illustration of EMG recording. Bipolar electrodes are implanted bilaterally into the masseter and unilaterally into the digastric muscles

To install the measurement device on the animal model, the mice received general anesthesia by intra-peritoneal injection of 5:1.5:3.5 ketamine (Ketalar; Sankyo Yell, Tokyo, Japan), xylazine (Selactar 2%; Bayer Health-care, Osaka, Japan), and 0.9% sodium chloride solution. The marker assembly was bonded to the lower surface of the mandible using 4-META resin (Super-Bond; Sun Medical, Moriyama, Japan). Simultaneously, bipolar electrodes consisting of non-stick coated stainless steel wires with 2-mm exposed tips and 1-mm interpolar distance were implanted bilaterally into the superficial masseter muscle and unilaterally in the anterior belly of the digastric muscle for recording EMGs, as shown in Fig. 2. Electrode wires were passed under the facial skin and fixed to a head connector, which was to be fixed on the top of the head.

After surgical preparation, the mouse was allowed to recover for 3 days. The head of the mouse was then firmly attached to the fixation device, and then mandibular movements were recorded at a frame rate of 200 frames/s. Simultaneously, EMGs of bilateral masseter muscles and the right digastric muscle were recorded. EMG signals were amplified with AC amplifiers and stored in a personal computer memory via a 16-bit analog-to-digital converter at a sampling frequency of 1000 Hz and processed using a high-pass filter with a 100-Hz cutoff frequency. At that time, EMG activities of masticatory muscles were recorded synchronously with mandibular movements using data acquisition software (Dipp-ADII; DITECT).

After optoelectronic measurement of mandibular movement using the motion capture system and EMG recording of masticatory muscle activity, CT images of the maxillofacial structure including the marker assembly were taken using an in vivo micro-CT system (R-mCT; Rigaku, Tokyo, Japan) to reconstruct the 3D anatomical model. The 3D surface of the maxillofacial bones including the four target markers attached to the mandible was reconstructed from CT images using 3D image processing and editing software (Mimics 20.0; Materialise, Leuven, Belgium). Through this procedure, the relative positions of maxillofacial bones with respect to the four markers were recorded.

**Fig. 3:**
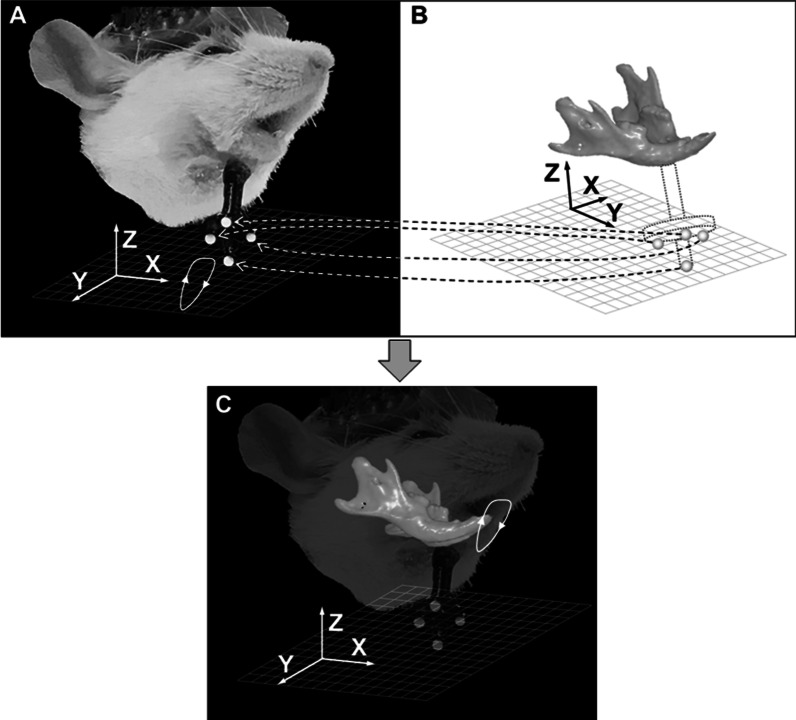
Diagram of the rigid transformation in which each of the coordinates of the four markers in the coordinate system of the anatomical model is transferred to that in the coordinate system of the motion capture system. **a** Coordinate system of the motion capture system. **b** Coordinate system of the anatomical model. **c** Anatomical data of the mandible are transferred to each motion capture image by registration of kinematic and anatomical data on the four markers

To reproduce and visualize mandibular movements, kinematic data obtained from the motion capture system were combined with anatomical data created from the micro-CT system. That is, surface images of the mandible were transferred to the motion capture images of mandibular movements. Since the coordinate systems of the motion capture system and anatomical model were not identical, coordinate transformation was performed by registration of kinematic data for mandibular movements and anatomical data of the mandible on four markers (Fig. 3). The coordinate transformation method has been previously described [12].

**Fig. 4:**
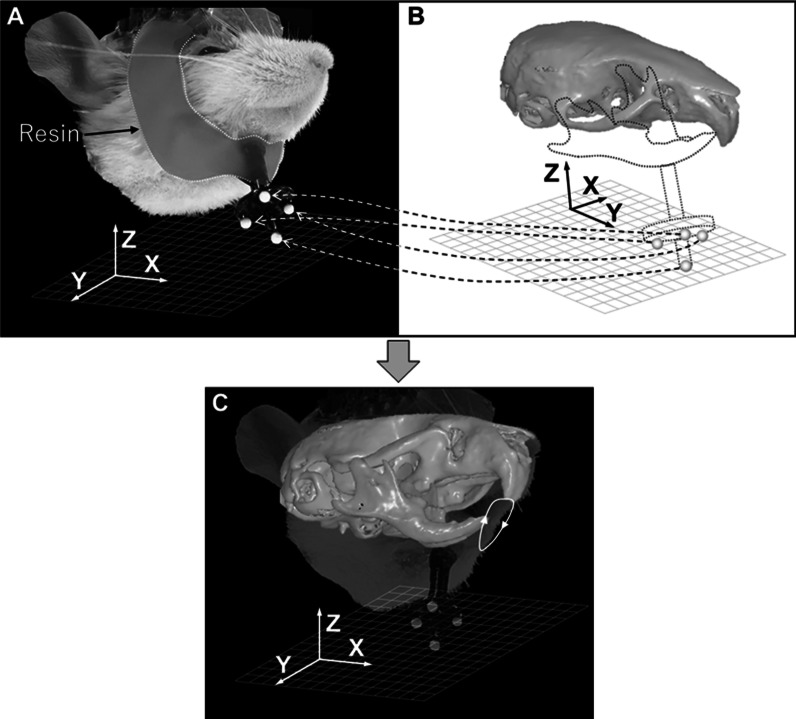
Diagram for visualization of mandibular movements relative to the maxilla. **a** A motion capture image representing the position of the entire maxillofacial structure with respect to four markers when the mandible is closed into an intercuspal position. **b** A micro-CT image representing the positional relationship between the maxilla with the adjacent bone structure and four markers. Transformation matrix and vector are calculated by registration of a motion capture image and a micro-CT image to the four markers. **c** The 3D reconstructed image of the maxilla with the adjacent facial and cranial complex is transferred to each frame of motion capture images of mandibular movement

To reproduce mandibular movements relative to the maxilla and hence to visualize and assess the status of occlusal contact between the upper and lower dentitions during mastication, the anatomical data of the upper dentition should be integrated with the kinematic data of mandibular movements with high accuracy. In other words, the 3D surface images of the maxilla, including the upper dentition, were transferred to motion capture images in the following procedure. The mouse was sacrificed while remaining attached to the head fixation device. At that time, the mouse received overdose of anesthesia for euthanasia by intra-peritoneal injection of ketamine (0.08 mg/kg) and xylazine (0.04 mg/kg). Next, the mandible was closed to an intercuspal position, and fixed together with the facial and cranial complex including the maxilla using 4-META resin to consolidate the two jaw bones into one unit so that the relative positions between them would remain unchanged (Fig. 4). The relative position of the maxillofacial structure with respect to the four markers was then precisely recorded by obtaining 3D images from both the motion capture system and the micro-CT system at the same jaw position. After recording two kinds of static images, coordinate transformation parameters between the two coordinate systems (and hence a transformation equation) were obtained in the same manner as already described. The 3D coordinates of all points on the surface of the maxilla with the surrounding maxillofacial structures were then inputted into the equation for transformation. Thus, 3D reconstructed images of the maxilla with the adjacent facial and cranial complex obtained from the micro-CT system were precisely superimposed onto each frame of the motion capture images. Dynamic transition of occlusal contacts between the upper and lower teeth associated with mandibular movements during mastication could thus be graphically visualized.

**Fig. 5:**
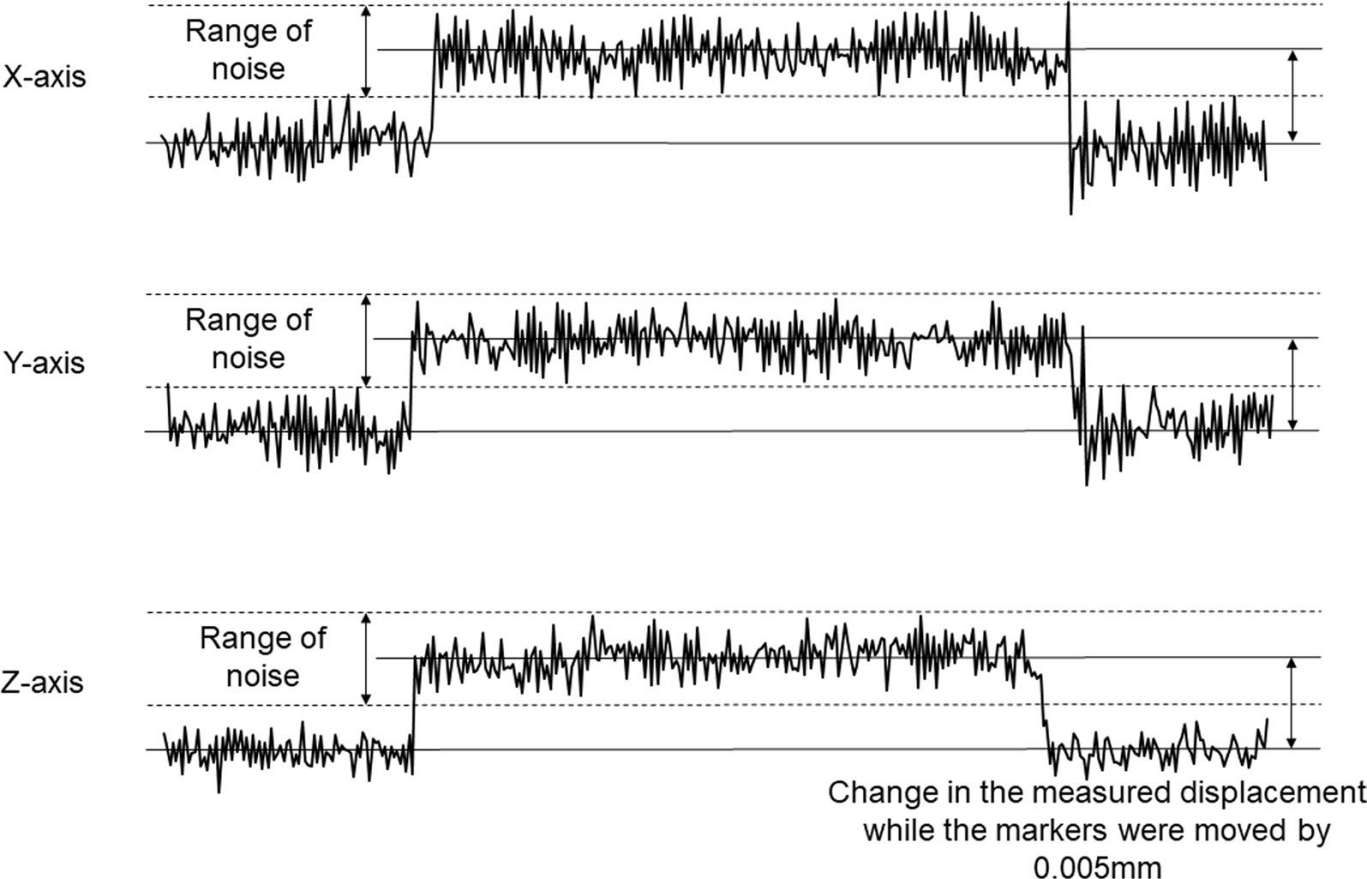
Resolution of the system for displacement. The marker assembly is moved along the x-axis (top), y-axis (middle), and z-axis (bottom) separately by 0.005 mm

**Fig. 6:**
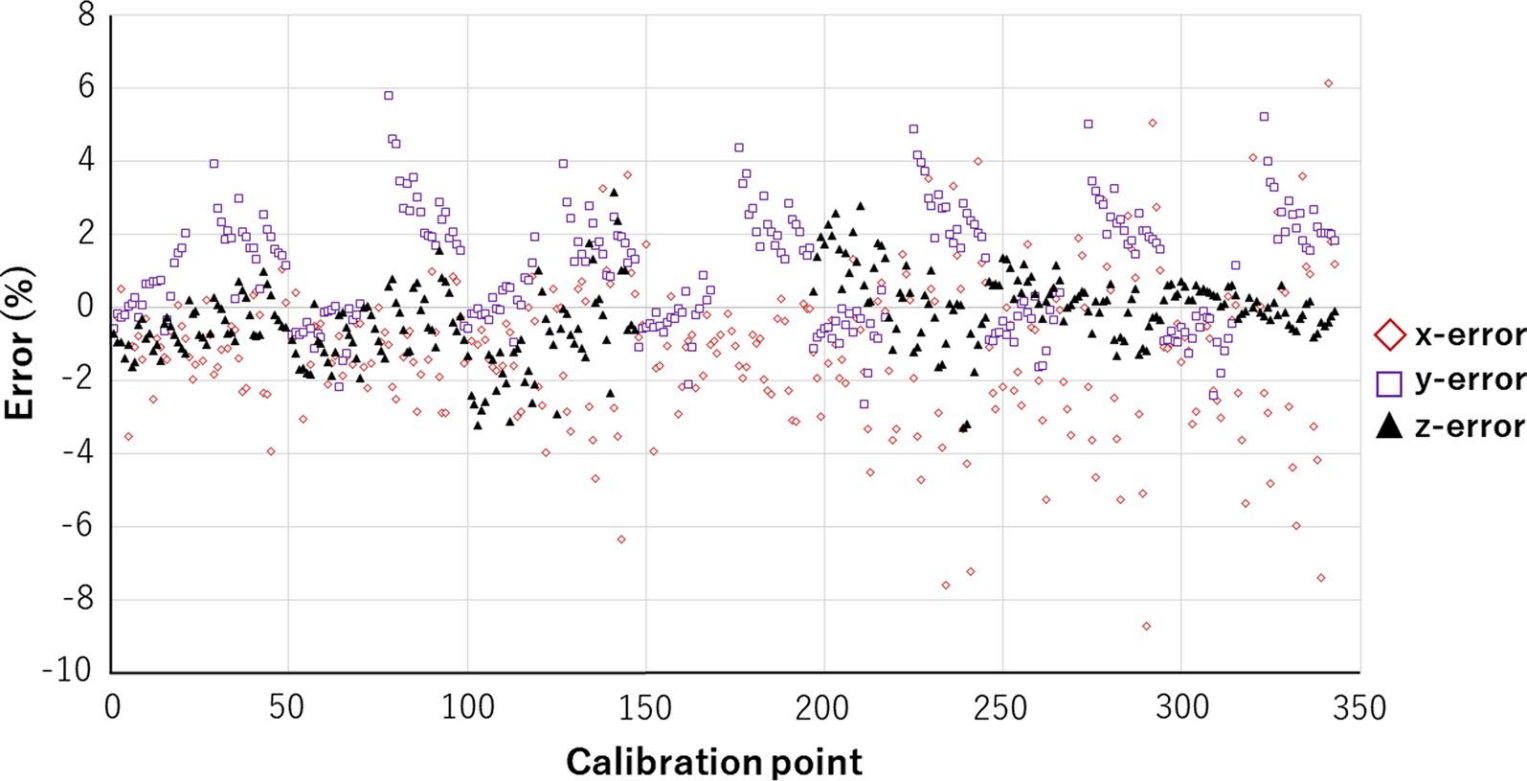
Measurement error at each calibration point

For system evaluation, the marker assembly was fixed onto a three-axis motorized pulse stage (MM-60XY, MM-60V; Chuo Precision Industrial Co., Tokyo, Japan). To determine the spatial resolution, displacement fluctuation was recorded along a time axis while the target marker on the stage was moved along the x-, y- and z-axes separately by 0.005 mm. Resolution was found to be less than 0.005 mm for the x-, y- and z-axes, since the change in displacement was larger than the range of the displacement fluctuation in all axes (Fig. 5). To determine the accuracy of the system, the target marker on the pulse stage was moved within the working area of 3 mm × 3 mm × 3 mm in 0.5-mm increments in 3D space. The difference between the displacement mechanically driven by the pulse stage and the measured displacement using the motion capture system was calculated at each of 343 driven points as the measurement error. Mean measurement errors were within 1.8% in the x-axis, 1.5% in the y-axis, and 0.8% in the z-axis (Fig. 6).

## Results

**Fig. 7:**
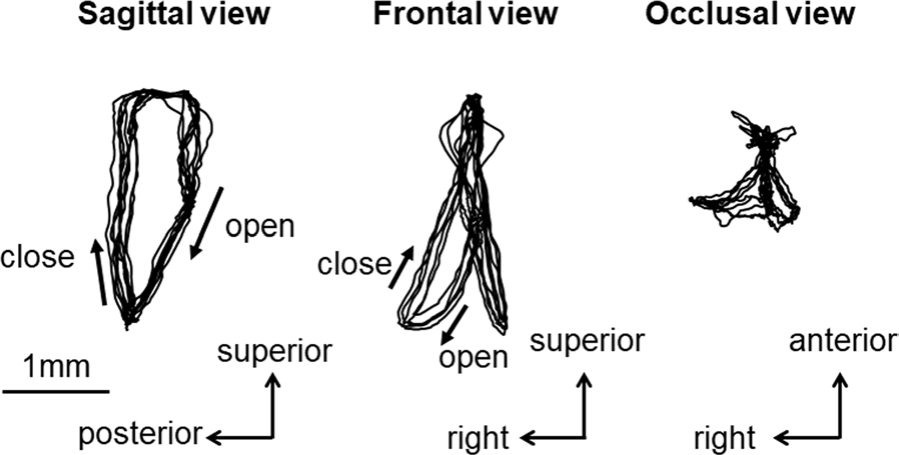
Movement trajectories of the molar projected on sagittal, frontal and occlusal planes during ten successive chewing strokes

In the first step, 3D movement trajectories on an arbitrary point on the mandible during mastication were reproduced by integrating the kinematic data of mandibular movements with the anatomical data of the mandible. As an example, movement trajectories of the molar during ten successive chewing strokes were reconstructed and projected on the sagittal, frontal, and occlusal planes, respectively (Fig. 7).

**Fig. 8:**
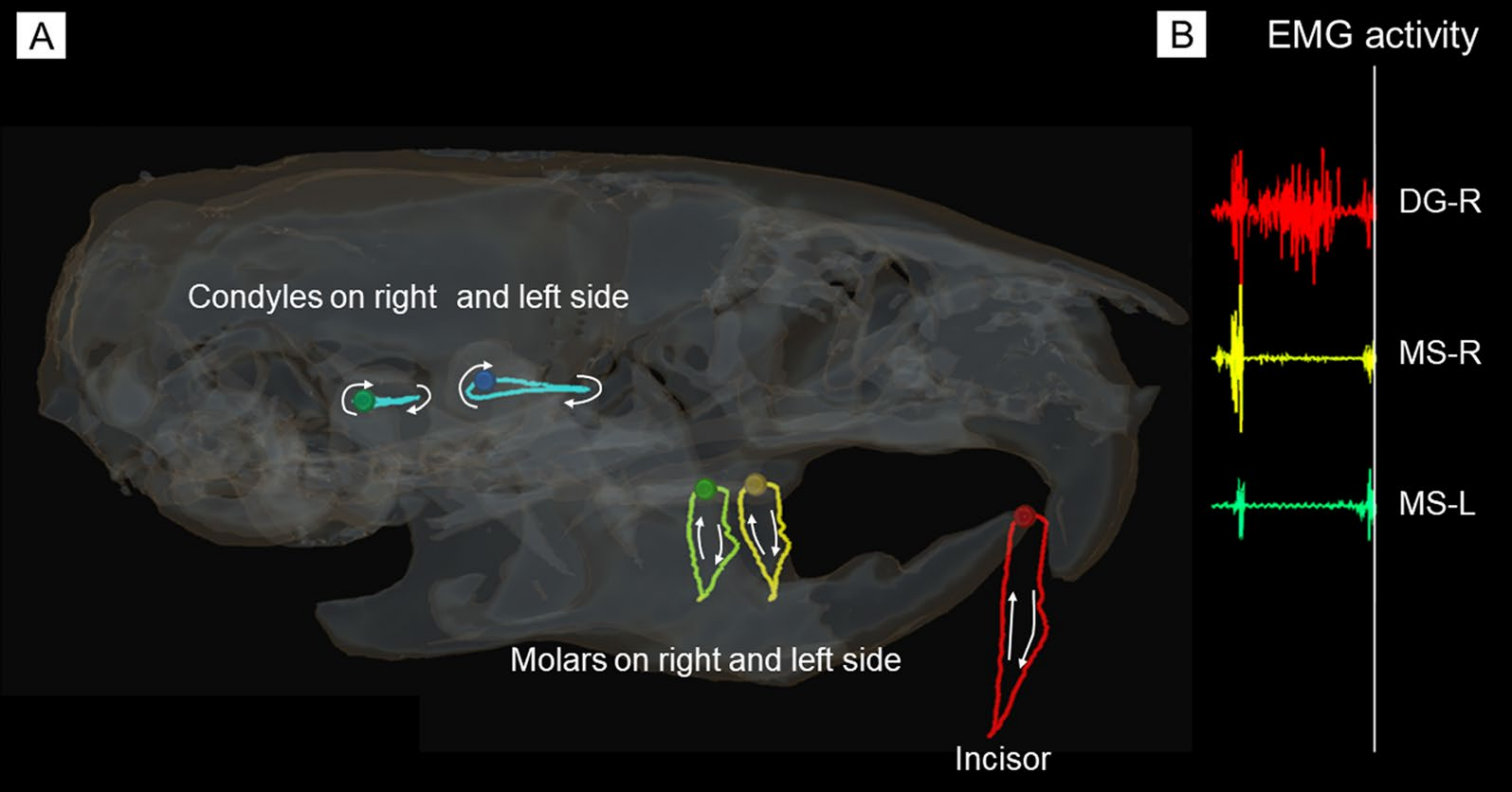
Paths of mandibular movements relative to the upper jaw anatomy from the lateral view and the corresponding EMG signals generated from masticatory muscles. A) Movement trajectories on analyzed points of the incisor, molars and condyles on right and left sides are shown. B) EMGs of masseter and digastric muscles recorded synchronously with mandibular movements. The vertical line indicates the timing of EMG on the corresponding jaw position

**Fig. 9:**
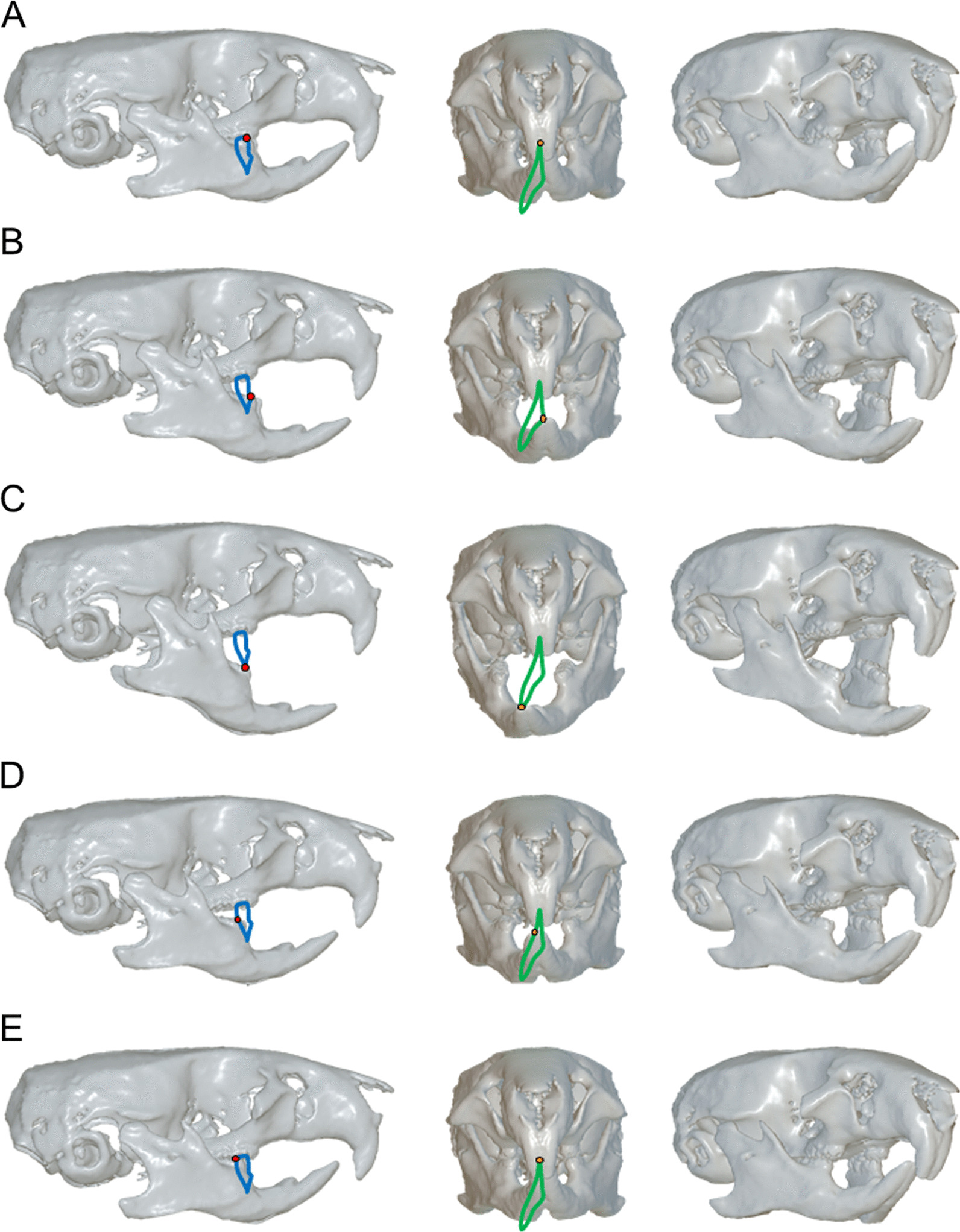
The 3D images of mandibular positions relative to the upper jaw anatomy corresponding to each timepoint. **a** Timepoint 1; **b** Timepoint 9; **c** Timepoint 19; **d** Timepoint 25; **e** Timepoint 28. Movement orbits of the lower molar (lateral view) and those of the lower incisor (frontal view) are indicated in blue and green, respectively. The mesiobuccal cusp of the lower molar and the incisal edge of the lower incisor are indicated by red and orange circles, respectively

**Fig. 10:**
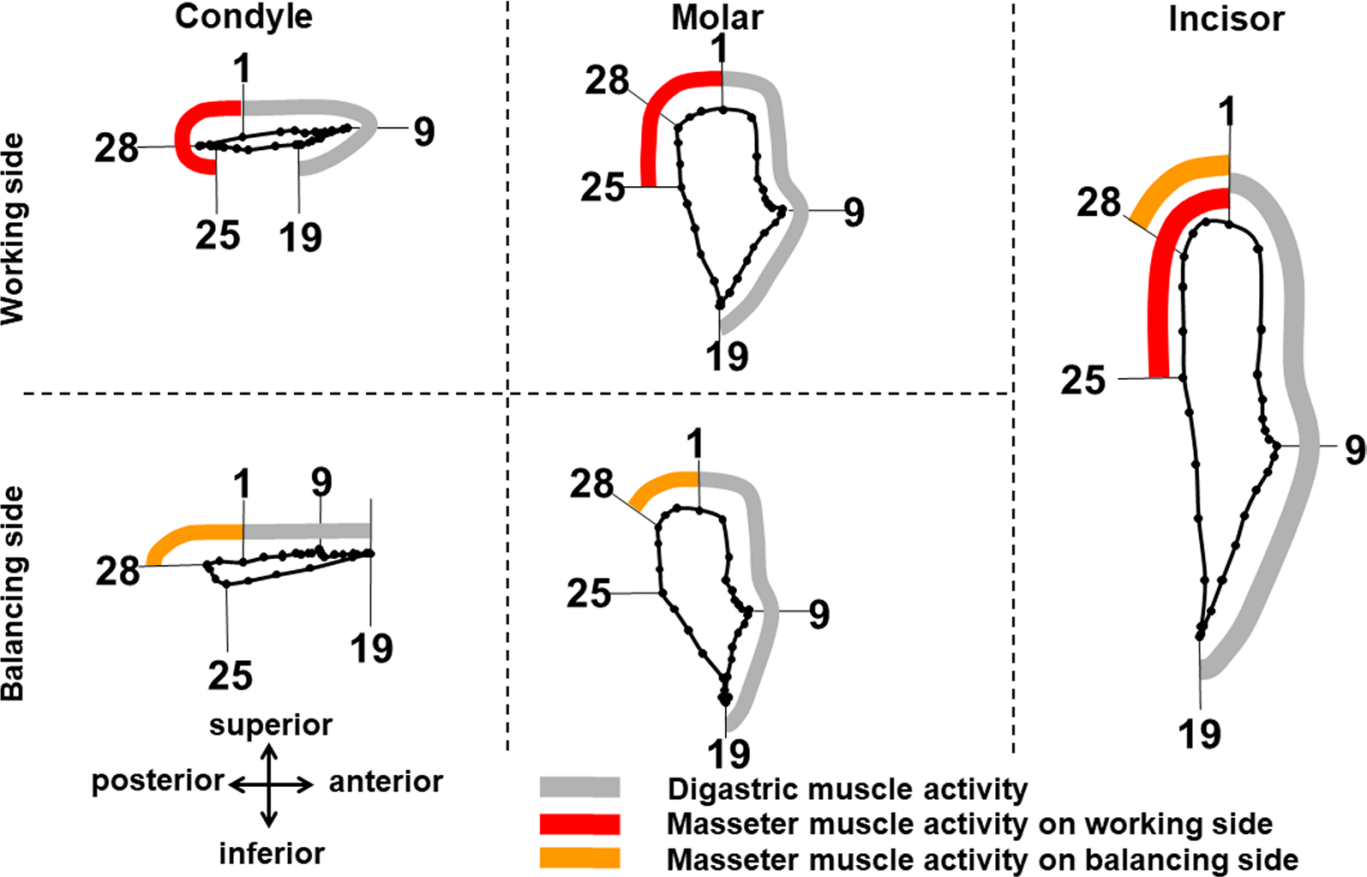
Movement trajectories of the incisor, molars and condyles on the working and balancing sides from the lateral view. The range of activities of the digastric muscle is plotted along the curvature of a typical movement orbit of each analyzed point in gray, and those of masseter muscles on the working side and balancing side in red and orange, respectively. Each small dot on the movement trajectories represents the position of an analyzed point for each frame of motion capture data. Thirty dots were plotted along the path of one chewing cycle with an interval of 5 ms

In the second step, 3D surface images of the maxilla including the upper dentition were transferred to each of the motion capture images. Mandibular movements relative to the maxilla, which involved dynamic occlusion representing the state of occlusal contacts between the upper and lower dentitions during mastication, were then reproduced and visualized. Figure 8a shows lateral view of mandibular movement paths relative to the upper jaw anatomy, linked to each analyzed point of the incisor, molars and condyles on the working and balancing sides. Figure 8b shows EMGs of the masseter and digastric muscles recorded synchronously with mandibular movements. Figure 9 shows 3D mandibular positions from lateral, frontal and right oblique views at each timepoint. Figure 10 shows the range of masticatory muscle activities associated with movement trajectories of the incisor, molars and condyles on the working and balancing sides projected on the sagittal plane.

Based on the movement trajectory of the molar, we determined that the mandibular position at timepoint 1 was the maximum jaw-closing position, that at timepoint 9 was the most anterior position, that at timepoint 19 was the maximum jaw-opening position, and that at timepoint 28 was the most posterior position, which coincided with the starting point of the occurrence of occlusal contacts between the upper and lower molars. According to the definitions of those timepoints, the masticatory cycle was divided into three phases: opening (timepoints 1–19); closing (timepoints 19–28); and occlusal (timepoints 28–1). Based on EMG signals, we determined that the mandibular position at timepoint 25 was the onset of activity in the masseter muscle on the working side, and timepoint 28 indicated the onset of activity in the masseter muscle on the balancing side, coinciding with the beginning of the occurrence of occlusal contact.

Movement trajectories of the incisor showed antero-posterior excursions unlike those of humans or other animals reported to date. The incisor traced a more posterior path in the closing phase (timepoints 19–28) than in the opening phase (timepoints 1–19). Movement trajectories of the condyle showed anterior and posterior movements that were nearly straight and parallel to the occlusal plane. In the opening phase, which corresponded to the duration of digastric muscle activity, the condyle on the working side moved anteriorly until the most anterior point (timepoint 9). Direction of movement then changed from anterior to posterior midway through the opening phase, and the condyle moved posteriorly up to the maximum jaw-opening position (timepoint 19). On the other hand, the condyle on the balancing side moved anteriorly throughout the opening phase. In the closing phase, the condyle on the working side further moved posteriorly from timepoint 19 halfway through the backward movement to the most posterior position of the condyle (timepoint 28). The condyle on the balancing side moved posteriorly from the anterior to the posterior border of the condylar movement path. In the occlusal phase, condyles on both working and balancing sides moved anteriorly from the most posterior position of the condyle (timepoint 28) to the maximum jaw-closing position (timepoint 1).

Regarding EMG activities of masseter muscles in association with condylar movement, a difference in duration existed between masseter muscle activities on the working side and those on the balancing side. That is, the onset of masseter muscle activities on the working side (timepoint 25, corresponding to the point slightly anterior to the most posterior position of the condyle) was earlier than that on the balancing side (timepoint 28, corresponding to the most posterior position of the condyle). The offset of masseter muscle activities was timepoint 1 on both working and balancing sides.

## Discussion

In this study, we have presented a novel method for visualizing mandibular movements relative to the upper dentition in mice based on analysis of stomatognathic function and reconstruction of a 3D model of the maxillofacial structure. That is, mandibular movements with six degrees of freedom were recorded using the motion capture system and the resulting kinematic data were applied to a 3D model of the anatomical structures created from micro-CT images. Masticatory muscle activities were recorded synchronously with measurements of mandibular movements.

Most previous investigations into oral motor behaviors using small animal models have attempted to detect movements of only one marker projected from the mandible, using a light-emitting diode in an optoelectronic measurement system [1, 3, 7, 29] or a magnet in an electromagnetic measurement system [8–10, 30–33] as a target marker to investigate the characteristics of mandibular movements. Since the mandible moves with six degrees of freedom during mastication, measurement of the movements of only one target point could never elucidate the entirety of mandibular movements. Using four markers in the motion capture system allowed practical measurement of mandibular movements with six degrees of freedom.

To reproduce movement trajectories for an arbitrary point on the mandible, we first employed a motion capture system comprising two high-speed cameras and four reflective markers to obtain the kinematic data. A 3D model of the mandible including markers was then reconstructed from micro-CT images, and movement trajectories on anatomical landmark points on the mandible such as incisor, molars and condylar points were reproduced by integrating the kinematic data of mandibular movements with the anatomical data of the mandible. Finally, 3D surface images of the upper dentition with the surrounding maxillofacial structures were transferred to each of motion capture images to reproduce mandibular movements relative to the upper jaw. This allowed visualization of dynamic occlusal contacts between the upper and lower dentitions during mastication. In addition, we recorded EMG activities of masticatory muscles associated with mandibular movements.

Compared to previously reported methods, the proposed system offers several key advantages in the evaluation of stomatognathic functions. First, the system could reproduce and visualize the movements of internal anatomical points such as condylar points by superimposing the micro-CT images of the mandible on each motion capture image. Second, the system could visualize and assess the status of occlusal contact between the upper and lower teeth during mastication by transferring 3D surface images of the upper dentition with the adjacent facial and cranial complex to each frame of motion capture images. In addition, the system could secure data synchronicity between jaw tracking and EMG recording, allowing elucidation of the role of masticatory muscles associated with mandibular movements.

As for system evaluation, displacement resolution was found to be less than 0.005 mm in each of the x-, y- and z-axes. Mean percentages of measurement errors were within 1.8% in the x-axis, 1.5% in the y-axis, and 0.8% in the z-axis of displacements in a working volume of 3 mm × 3 mm × 3 mm in 0.5-mm steps. The accuracy of the system was thus considered to be extremely high as compared with previously described systems [8, 29, 34, 35].

Since the occlusal contact associated with mandibular movement could be visualized, the occlusal phase was identified as the duration from the beginning to the end of the occurrence of occlusal contacts between the upper and lower molars on the basis of the movement trajectories of the molars. Consequently, the masticatory cycle was found to be clearly divisible into opening, closing, and occlusal phases.

The most important feature of the system was the capability to record EMG activities of masticatory muscles synchronously with mandibular movements. Thanks to this capability, we clarified that coordinated activation of the right and left masseter muscles plays an important role in forming a unique path of mandibular movement in mice. In the opening phase, the condyles on both sides moved anteriorly, then the condyle on the working side moved back in the midway, while that on the balancing side continued to move anteriorly until the end of the opening phase (Fig. 10). Accordingly, the mandible was displaced laterally to the right or left side during jaw-opening. In the closing phase, right and left condyles moved posteriorly and were ultimately retracted into the most posterior and a more symmetrical position, corresponding to the beginning of the occlusal phase. Regarding the activities of masticatory muscles associated with condylar movements, a discrepancy in onset of masseter muscle activities was evident between right and left. The onset of activity in the masseter muscle on the working side was significantly earlier than that on the balancing side. That is, activity of the masseter muscle on the working side began in the latter half of the closing phase, while that on the balancing side began at the starting point of the occlusal phase. A previous study [10] reported that the temporalis muscle tends to retract the mandible posteriorly from the beginning of the closing phase, and the masseter muscle later tends to pull the mandible forward from the middle of the closing phase, since the temporalis muscle has a more posteriorly oriented force vector, while the masseter muscle has a more anterior one. These findings therefore suggest that in the latter half of closing phase, earlier onset of activity in the masseter muscle on the working side could slow posterior movement of the condyle on that side until the condyle on the balancing side catches up on the posterior movement with the condyle on the contralateral side. Condyles on both sides then moved into the same position, which was the most posterior position, and anterior movement (namely, the occlusal phase) could be started from the symmetrical position of the right and left condyles. These findings indicated that a characteristic feature of condylar movements in mice is formed by the regulation of motor coordination between the right and left masseter muscles. The proposed system has the potential to elucidate the mechanism of motor coordination of masticatory muscles and their roles during mastication by taking advantage of the capability to record EMG data synchronously with mandibular movements.

Future studies may incorporate quantification of occlusal contacts, namely, clearance between the upper and lower teeth and joint space between the condyle and glenoid fossa, which could estimate the intensity of mechanical loading acting on the corresponding area and the risk of causing orofacial myofunctional disorder such as temporomandibular disorder. If real-time EMG recording of several more masticatory muscles other than masseter and digastric muscles could be performed, the pathogenetic mechanisms of oral disorder associated with mechanobiological dysfunction could be clarified using animal models. The current approach enables simultaneous analyses of morphology and function of the jaws and face, and hence the system is considered to be useful for elucidation of pathophysiology of temporomandibular disorder caused by degenerative changes of the mandibular condyle, or Parkinson's disease and muscular dystrophy caused by neuromuscular disorder. Full investigation into stomatognathic function in genetically modified mice such as models of temporomandibular joint osteoarthritis [36, 37] and oral dyskinesias [20, 38] would greatly enhance our understanding of the etiology and diagnosis of neural and motor disorders in the craniomandibular system.

## Conclusions

The proposed system could measure mandibular movements with six degrees of freedom and visualize the movements of internal anatomical points such as condylar points with high accuracy. This was achieved by integrating kinematic data obtained from the motion capture system with geometric data of the mandible from micro-CT images. Further, dynamic transition of occlusal contacts between the upper and lower dentitions could be visualized by superimposing the 3D reconstructed image of the maxilla onto motion capture images of mandibular movements. The ability to record EMG activities of masticatory muscles synchronously with mandibular movements allowed elucidation of the mechanisms of motor coordination for these muscles and their roles during mastication. Deeper insights into the analysis of stomatognathic function using such systems will lead to clarification of the pathogenesis and diagnosis of various oral dysfunctions.

## Data Availability

Data of the present study will be shared upon request to the corresponding Author.

## Abbreviations

3D: 3-Dimensional
EMG: Electromyography
micro-CT: Micro-computed tomography
